# Effects of environmental radiation on testes and spermatogenesis in wild large Japanese field mice (*Apodemus speciosus*) from Fukushima

**DOI:** 10.1038/srep23601

**Published:** 2016-03-23

**Authors:** Tsukasa Okano, Hiroko Ishiniwa, Manabu Onuma, Junji Shindo, Yasushi Yokohata, Masanori Tamaoki

**Affiliations:** 1 Ecological Genetics Analysis Section, Center for Environmental Biology and Ecosystem Studies, National Institute for Environmental Studies, 16-2 Onogawa, Tsukuba, Ibaraki 305-8506, Japan; 2 Laboratory of Wildlife Science, School of Veterinary Medicine, Kitasato University, Higashi 23-35-1, Towada, Aomori 034-8628, Japan; 3 Graduate School of Science and Engineering, University of Toyama, 3190 Gofuku, Toyama, Toyama 930-8555, Japan

## Abstract

The Fukushima Daiichi Nuclear Power Plant (FDNPP) accident that occurred after the Great East Japan Earthquake in March 2011 released large quantities of radionuclides to the environment. The long-term effects of radioactive cesium (Cs) on biota are of particular concern. We investigated the accumulation of radioactive Cs derived from the FDNPP accident, and chronic effects of environmental radionuclides on male reproduction, in the large Japanese field mouse (*Apodemus speciosus*). In 2013 and 2014, wild mice were captured at 2 sites in Fukushima Prefecture and at 2 control sites that were distant from Fukushima. Although the median concentrations of ^134^Cs and ^137^Cs in the mice from Fukushima exceeded 4,000 Bq/kg, there were no significant differences in the apoptotic cell frequencies or the frequencies of morphologically abnormal sperm among the capture sites. Thus, we conclude that radiation did not cause substantial male subfertility in Fukushima during 2013 and 2014, and radionuclide pollution levels in the study sites would not be detrimental to spermatogenesis of the wild mice in Fukushima.

The Fukushima Daiichi Nuclear Power Plant (FDNPP) accident that occurred after the Great East Japan Earthquake in March 2011 released approximately 500 PBq of ^131^I, 10 PBq of ^134^Cs, and 10 PBq of ^137^Cs to the environment^1^. ^137^Cs has a relatively long half-life of approximately 30 years; therefore, the long-term effects of its gamma ray emissions on biota (such as wild animals) are of particular concern. The United Nations Scientific Committee on the Effects of Atomic Radiation (UNSCEAR) reported that chronic dose rates less than 100 μGy/h would not have a significant effect on most terrestrial communities^2^. In addition, the International Commission on Radiological Protection (ICRP)^3^ reported that there is a very low probability that dose rates of 0.1–1.0 mGy/day (4.2–41.7 μGy/h) would affect rodents. To date, there have been some reports of biological effects on wildlife caused by radioactive materials from the FDNPP accident. For example, previous studies have shown morphological abnormalities in the pale grass blue butterfly (*Zizeeria maha*)^4^, reduced abundance of common birds^5^, low blood cell counts in wild Japanese monkeys (*Macaca fuscata*)^6^, and reduced reproductive performance in the goshawk (*Accipiter gentilis*)^7^.

Rodents are considered to be a suitable model for studying ecological effects of radiation on wild animals because they have high fertility and short generation times, and because they inhabit the top layers of the soil where the highest doses of radiation are detected^8^. Furthermore, reference data from experiments on rodents are abundant, and the ICRP^3^ selected rodents as reference animals for environmental protection studies. Therefore, we selected the large Japanese field mouse (*Apodemus speciosus*) for our investigation of radiation effects in this study.

The large Japanese field mouse is a common rodent with a range that includes almost all of Japan (from Hokkaido to Kyusyu). They inhabit a wide range of environments, including forests, plantations, riverside fields, and cultivated fields^9^. In Fukushima, as in most of the Japanese mainland with the exception of highlands, they typically breed twice yearly, in spring and autumn^10^,^11^,^12^. Their maximum life span in the wild is estimated to be 26 months^13^. Their main diet includes the roots and stem of herbaceous plants, seeds, berries, and insects^14^. In addition, field mice are important prey for medium-sized predators, including foxes, martens, and owls^15^,^16^.

Testes are one of the most sensitive organs to radiation^17^,^18^, and heritable abnormalities induced in germ cells or gametes are of special interest. Organisms within the zone of greatest atmospheric deposition (25–45 km northwest from FDNNP) were predicted to be exposed to considerable radiation for the first 30 days after the accident; forest rodents were exposed to 3.9 mGy/day during that period^19^. This value is within the “reduced reproductive success” level, which ranges from 1 to 100 mGy/day for rodents^3^.

Therefore, in this study we investigated the accumulation of radioactive Cs derived from the FDNPP accident in large Japanese field mice, and the chronic effects of environmental radionuclides for male reproduction, focusing on germ cell apoptosis and sperm morphology. Wild large Japanese field mice were captured at 2 sites in Fukushima Prefecture (Sites 1 and 2) and from 2 other control sites (Sites 3 and 4; Fig. 1). The results are expected to provide scientific information for biological and ecological risk assessments involving environmental radioactive Cs.

## Results

### Ambient dose rate and Cs accumulation in animals and soil

The ambient dose rates at wild rodent capture sites are shown in Table 1. “Ambient dose rate” is defined as the gamma ray energy absorbed by a mass of ambient per unit time. The ambient dose rates at Site 1 were not significantly different between 2013 and 2014 (U-test, *P* = 0.835). In contrast, the ambient dose rates at Site 2 decreased significantly between 2013 and 2014 (U-test, *P* = 0.037). The ambient dose rates at Site 1 were significantly higher than those at Site 2 in each year (2013 and 2014, U-test, *P* = 0.012).

Concentrations of ^134^Cs and ^137^Cs radioactivity in the soil and in large Japanese field mice are shown in Tables 2 and 3. Considerable differences among soil and mouse samples from the same site were observed. The average radioactivity concentrations of soils from Sites 1 and 2 were more than 100,000 Bq/kg every year, but those from Sites 3 and 4 were less than 30 Bq/kg. There were significant differences in the soil concentrations of radioactivity among different sites in the same year (2013, U-test, *P* = 0.060; 2014, Kruskal-Wallis test, *P* = 0.0002). However, there were no significant differences between 2013 and 2014 in the soil radioactivity concentrations at Sites 1 and 2 (U-test; Site 1, *P* = 1.000; Site 2, *P* = 0.296).

The median concentrations of ^134^Cs and ^137^Cs radioactivity for mice from Sites 1 and 2 were more than 4,000 Bq/kg, but concentrations were 0 Bq/kg for mice from Sites 3 and 4. For each year, there were significant differences among sites in mouse radioactivity concentrations (Kruskal-Wallis tests: 2013, *P* < 0.0001; 2014, P < 0.0001). However, significant changes in mouse radioactivity concentrations were not observed between 2013 and 2014 at any examined site, although the total Cs concentrations in mice from Fukushima sites increased non-significantly (U-test; Site 1, *P* = 0.106; Site 2, *P* = 0.051; Site 3, *P* = 0.500; Site 4, *P* = 0.960).

### Frequency of apoptosis in germ cells

Apoptotic germ cells were observed in seminiferous tubules of all mice (Fig. 2a,b). The frequency of apoptotic cells did not differ between the Fukushima sites and the control sites, and it showed no change between capture years (Table 4; Kruskal-Wallis test, sites and years together, *P* = 0.131). Furthermore, seminiferous tubule diameter, which is affected by apoptotic cell death, did not vary with either capture site or capture year (Table 4; Kruskal-Wallis test, sites and years together, *P* = 0.582).

### Frequency of abnormal sperm morphologies

The median frequency of morphologically normal sperm ranged from 78% to 85%, and there were no differences in the frequency of normal sperm cells among capture sites and/or between capture years (Fig. 2c,d; Table 5; Kruskal-Wallis test, sites and years together, *P* = 0.596). In addition, the frequencies of morphological abnormalities of the sperm head, midpiece, and tail also showed no significant differences among capture sites and/or capture years (Table 5; Kruskal-Wallis test, sites and years together; head, *P* = 0.373; midpiece, *P* = 0.568; tail, *P* = 0.557).

## Discussion

The majority of the radioactive materials derived from the FDNPP accident were deposited in the upper soil layer and plant litter in the forest^20^. Because *Apodemus speciosus* has low body height and inhabits the contaminated ground surface, it may have suffered higher exposure to external radiation sources than other mammals living in Fukushima. The average dose rate measured on the ground was approximately 1.4-fold higher than the dose rate 1 m above the ground^21^. The average ambient dose rate of gamma rays at ground level in the Fukushima sites was 4.1–13.9 μSv/h. This level is the same as the “very low probability of effects” level in the ICRP criteria^3^. Furthermore, *Apodemus speciosus*, being omnivorous, could feed on soil invertebrates that showed high levels of radioactive material^22^. Therefore, it may have suffered from higher internal exposures than herbivores experienced.

The ambient dose rates at Site 2 decreased significantly from 2013 to 2014, but the radioactive Cs concentrations of the soil from Site 2 increased non-significantly from 2013 to 2014. Similar trends were also observed in Site 1. One might expect that ambient dose rates positively correlate with radioactive Cs concentrations of the soil. However, disparities were observed between the ambient dose rates and the soil Cs concentrations. The contamination with radioactive Cs is not uniform across the field^23^. Non-uniform field contamination may have affected the soil values because of small sample numbers and weights, and because the sampled points differed among years.

There are many reports of Cs contamination of wildlife in eastern Japan after the accident, and the situation is becoming clear. In some species of wild birds and mammals in Fukushima, radioactive Cs decreased from April 2011 to the end of 2013. On the other hand, the species inhabiting forests tended to retain high levels of radioactivity^24^. Furthermore, radioactive Cs concentration levels differed among the species. For example, omnivorous animals such as Asian black bear (*Ursus thibetanus*) and wild boar (*Sus scrofa*) showed high concentrations of Cs, while Cs concentrations in herbivorous animals such as deer (*Cervus nippon*) and duck (e.g., *Anas poecilorhyncha*) were low^25^. A previous report showed that the Cs concentration of wild mice captured at Fukushima ranged from 870 to 8,040 Bq/kg wet weight 7 to 9 months after the accident^26^. In another study, mice captured at a severely contaminated site, where the ambient dose rate measured on the ground reached approximately 60 μSv/h in December 2013, exhibited Cs concentrations of 32,700 ± 23,200 (mean ± SD) Bq/kg wet weight in a mixture of bone and muscle^21^. Taken together, these results in wild mice showed high variation in Cs concentration even among mice captured at the same site.

It is thought that the large variation in Cs concentration among individual mice may come from differences in food utilization^14^. Indeed, our preliminary observations indicated that mice that showed higher Cs concentrations preferred to eat bamboo roots rather than conifer cones (Tamaoki *et al.* personal communication). In addition, the large Japanese field mouse can easily migrate through areas with different contamination levels^27^. Their migrations are affected by food availability, population size, geography, and breeding status^28^,^29^. Further studies on the relationship between Cs contamination and food utilization in mice are warranted.

Germ cell apoptosis is an informative marker of ionizing radiation and some other toxicants^30^,^31^. Intracellular reactive oxygen species generated by radiation are major inducers of apoptosis^32^. Loss of male germ cells by apoptosis, which has the potential to cause infertility, could be attributed to ionizing radiation^33^. On the other hand, selective removal of damaged germ cells by apoptosis is a very important mechanism for preventing the transmission of genetic abnormalities to offspring^34^. In this study, there were no harmful effects on germ cells from environmental radiation. Indeed, few apoptotic cells were detected in animals captured at any of the sites, even the highly contaminated Site 1. There have been few studies on radiation effects on male mammal fertility following the FDNPP accident; however, no effects on bull testes and sperm were observed within a 20-km zone around the FDNPP between August 2011 and January 2012^35^. In contrast, there are some reports about sperm morphology following the Chernobyl Nuclear Power Plant (CNPP) accident. House mice (*Mus musculus*) were captured between 1986 and 1994 within a 30-km zone around the CNPP, and the frequencies of abnormal sperm heads did not differ significantly among sites with different pollution levels^36^. Another study of 11 species of passerine birds caught in Chernobyl showed that frequencies of abnormal sperm were always higher in heavily contaminated areas of Chernobyl than in uncontaminated areas^37^. In laboratory mice, low-dose-rate (3.49 mGy/h) gamma ray exposure significantly decreased testes weights and seminiferous tubule diameters at 2,000 mGy total exposure, but a decrease was not observed at 20 to 200 mGy^38^. In addition, exposure to 25 to 250 mGy X-rays at a low-dose-rate (12.5 mGy/h) significantly increased male germ cell apoptosis, with a maximum effect at 75 mGy^33^. In that study, percentages of TUNEL positive germ cells and apoptotic spermatogonia were approximately 20% without irradiation, and approximately 60% with 75 mGy irradiation^33^. Radiation levels in these experiments were considerably higher than the ambient dose rates (under 0.02 mGy/h) at the wild mouse capture sites in Fukushima. Furthermore, it is thought that the effect of external radiation exposure on wild animals is likely to be greater than the effect of internal exposure in wild mammals^39^,^40^. Because loss of male germ cells was not detected in this study, we conclude that radiation has not caused substantial male subfertility in wild large Japanese field mice in Fukushima.

In this study, there were no significant differences in the frequency of morphologically normal sperm among wild mice captured at different sites. High dose and high-dose-rate irradiation disrupts spermatogenesis and increases the frequency of abnormal sperm^41^,^42^. Spermatozoa are produced through complex processes in the testes and epididymides, and defects in these processes can result in male infertility^43^. In addition, there is a positive correlation between DNA defects that might affect the next generation and altered sperm head morphology^44^. There have been a few reports that investigated whether low-dose irradiation causes altered sperm morphology. Following the Chernobyl accident, Møller *et al.* reported that the frequency of abnormal sperm in some bird species increased at highly radiation-contaminated areas^37^,^45^,^46^. In a laboratory test on ICR mice, low-dose-rate (0.7 mGy/h) gamma ray exposure did not affect sperm morphology at 200 to 4,000 mGy total radiation. However high-dose-rate (48 Gy/h) exposure with similar total radiation levels (from 200 to 4,000 mGy) increased sperm abnormalities significantly^47^. Another experiment, using Korean dark-striped field mice *(Apodemus agrarius*), showed similar results^48^. In contrast, increased rates of morphologically abnormal sperm were not observed in the wild mice captured in highly contaminated areas of Fukushima. Although the data do not indicate exactly how large a radiation dose the mice absorbed before they were captured, we conclude that low-dose-rate radiation at the environmentally observed level would not be detrimental to spermatogenesis.

Our study suggests that high levels of radioactive contamination can have surprisingly limited effects on spermatogenesis in some species. Although we expected field mice would receive higher radiation doses than most other animals, and we expected that testes would be especially sensitive to radiation, we found no evidence of detrimental effects on testes or sperm. Although our study did not measure fertility, it suggests that effects of the FDNPP accident on field mouse spermatogenesis are surprisingly weak. Given that effects were shown in previous studies on other taxa, and the high levels of within-site variation in contamination observed here, further studies are needed to investigate the roles of taxonomic differences in behavior, trophic level, and physiology on the effects of radiation exposure.

## Methods

### Animals and capture sites

**Figure 1:**
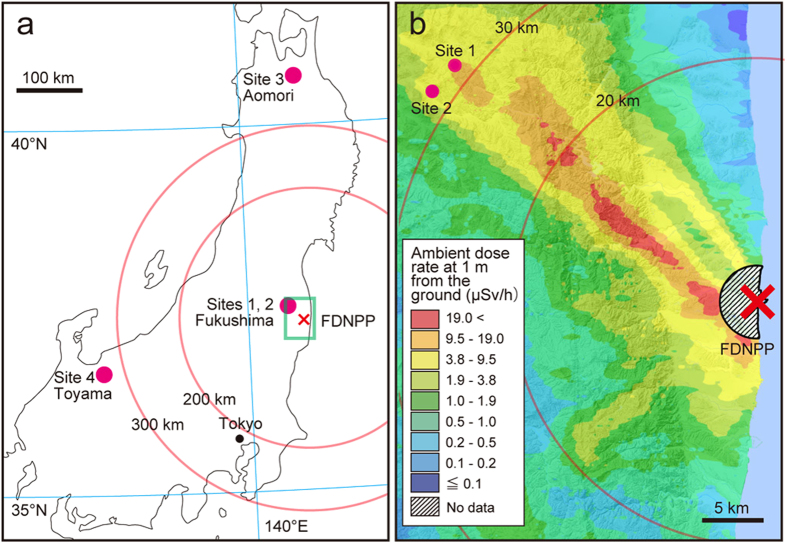
Capture sites for large Japanese field mice used for this study (**a**) and ambient dose rate 30 months after the Fukushima Daiichi Nuclear Power Plant (FDNPP) accident (**b**). The region in the green box in panel ‘**a**’ is shown enlarged in panel ‘**b**’. The map in panel ‘**a**’ was created using royalty-free digital map source software (MAPIO ’05-’06 nenndo-ban JAPAN, DesignEXchange, Tokyo, Japan). The map in panel ‘**b**’ with ambient dose rate data was created from ‘Extension Site of Distribution Map of Radiation Dose, etc.,/ Digital Japan’ (http://ramap.jmc.or.jp/map/eng/map.html, Accessed 4 Nov. 2015).

**Figure 2:**
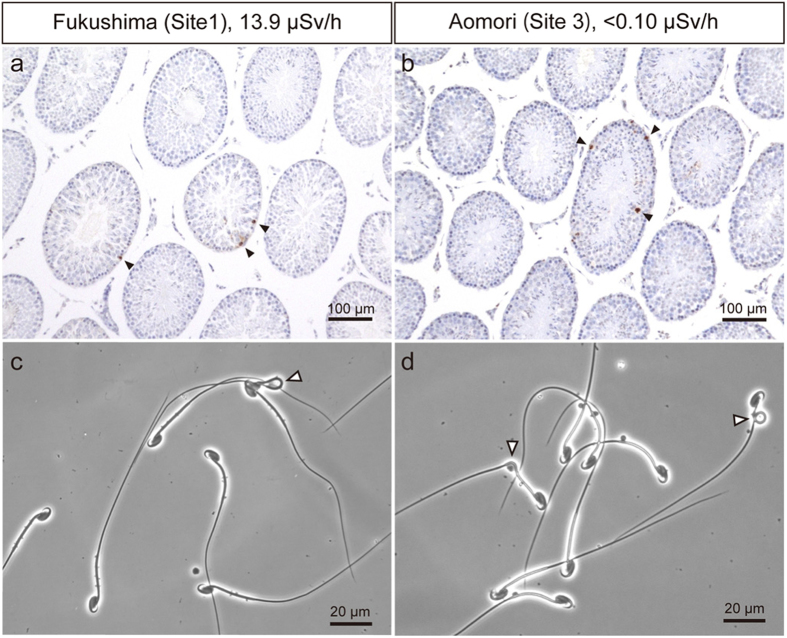
Apoptosis in male germ cells, and morphology of epididymal spermatozoa in wild large Japanese field mice captured at Fukushima (Site 1) and Aomori (Site 3) in 2013. (**a,b**) Terminal deoxynucleotidyl transferase-mediated dUTP nick end labeling (TUNEL) staining and hematoxylin counterstaining of seminiferous tubules (Solid arrowheads: Apoptotic cells). (**c,d**) Phase-contrast micrograph of spermatozoa (Open arrowheads: spermatozoa with abnormal morphologies).

Wild large Japanese field mice were captured using Sherman traps between July 2013 and September 2014 at 4 sites described below (Fig. 1). These sites were located different distances and directions from FDNPP (except for 2 sites within Fukushima, which showed different ambient dose rates). The sites had similar habitats, consisting mostly of temperate zone deciduous forest. These sites were as follows:

*Site 1*: Fukushima Prefecture, approximately 32 km northwest from FDNPP (37°36′N, 140°45′E; approximately 580 m elevation), the ambient dose rates were approximately 5-fold higher than those of Site 2; *Site 2*: Fukushima Prefecture, approximately 32 km northwest (37°34′N, 140°43′E; approximately 540 m elevation); *Site 3*: Aomori Prefecture, a control site approximately 350 km north (40°35′N, 140°57′E; approximately 390 m elevation); *Site 4*: Toyama Prefecture, a control site approximately 320 km southwest (36°35′N, 137°24′E; approximately 620 m elevation).

Adult male mice captured in the breeding season (April and July-September), with body weight over 30 g, and paired testes weight over 1.0 g were used for this study because sexually mature adult males have body weight over 30 g^10^ and adult males in the breeding season have paired testes weight over 1.0 g^12^.

### Ambient dose rate measurement

The ambient dose rates of gamma radiation were measured at ground level using a handheld survey meter (type: NHE20CY3-131By-S, Fuji Electric Co., Ltd., Tokyo) at Fukushima Sites 1 and 2 in November 2013 and August 2014. Large Japanese field mice are expected to be exposed to radiation mainly at ground level. In each site and year, dose rates were measured at 5 spots, which were over 10 m from each other. Measured spots were different every year, but were within the same area (30–40 m in radius) in every year.

### Soil collecting

Soil was collected from Sites 1 and 2 (Fukushima) in November 2013 and August 2014, and Sites 3 and 4 (control) during July-August 2014. For each site and year, soil samples were collected from the surface (0–1 cm depth) at the same 5 spots as the ambient dose rate measurements. Because the surface layer of soil are most contaminated by radioactive cesium^20^, radiocesium in soil could be measured effectively even in rarely contaminated sites. The samples, approximately 10–40 g wet weight, were enclosed in 100-mL polystyrene containers (50 mm in inner diameter, 62 mm in height) for Cs measurements.

### Mouse sample collecting

All experimental protocols were approved by the National Institute for Environmental Studies committee for the Great East Japan Earthquake and the Fukushima Daiichi Nuclear Power Plant accident research (project name: Long-term monitoring of the biological effects of radiation exposure on terrestrial wildlife. responsible researcher: Manabu Onuma). The mice were handled in a humane manner in accordance with the Mammal Society of Japan’s guidelines for using wild mammals^49^. Before sample collection, the captured mice were sacrificed by CO_2_ asphyxiation. Body weights were measured, and the testes and epididymides were removed surgically. Paired testes were weighed, and fixed in 10% neutral buffered formalin for assessment of germ cell apoptosis.

For collecting spermatozoa, the cauda of one epididymis was immersed in 200 μL physiological saline at room temperature. The spermatozoa were released by mincing the epididymis in the saline. After 3 min, a 50-μL sperm suspension was collected and gently mixed with an equal volume of fixative (2% v/v glutaraldehyde in 0.165 M sodium cacodylate/HCl, pH 7.3).

The bodies, minus the testes, epididymides, heads, intra-abdominal organs, and contents of the stomach and intestine, were minced using a food processer. Minced samples, approximately 20–30 g wet weight, were enclosed in 100-mL polystyrene containers for Cs measurements.

### Germ cell apoptosis

The fixed testes were dehydrated in graded ethanol, embedded in paraffin wax, and sliced into 4-μm sections. For detecting germ cell apoptosis, terminal deoxynucleotidyl transferase-mediated dUTP nick end-labeling (TUNEL) was performed with ApopTag Peroxidase *In Situ* Apoptosis Detection Kit (Merck Millipore, Darmstadt, Germany), following the manufacturer’s instructions. The sections were counterstained with hematoxylin, and examined under a light microscope (BZ-9000, KEYENCE, Osaka, Japan).

TUNEL positive (apoptotic) germ cells were counted in randomly selected seminiferous tubule cross-sections of 3–4 × 10^6^ μm^2^. The cross sectional areas were measured using the BZ-9000 analysis software. Male germ cell apoptosis frequencies were presented as the number of TUNEL positive cells divided by the cross-sectional area (in mm^2^).

In each mouse, the diameters (minor axes) of 20 randomly selected seminiferous tubules with round cross-sections were measured using the BZ-9000 analysis software. The average tubule diameter per mouse was calculated.

### Sperm morphology

The fixed sperm samples were placed on slides, and covered with cover glasses, which were sealed with nail polish. Morphologies of the head, midpoint, and tail of 100 randomly selected spermatozoa were examined using a phase-contrast microscope (BZ-9000). Morphologies were categorized as abnormal if they displayed the following features:

*Head*: double, amorphous, small, large, twisted hook, short hook, long hook, and straight hook; *Midpiece*: double, kinked, damaged, folded, clumped, thin, thick, short, hairpin-shaped, bent, and coiled; *Tail*: double, partially thin, short, twisted, tightly coiled, folded, hairpin-shaped, simply coiled, and bent.

### Cs accumulation in animals and soil

Prepared samples enclosed in 100-mL containers were used for analysis of ^134^Cs and ^137^Cs. The ^134^Cs and ^137^Cs activities of the samples were measured with high-purity germanium (HpGe) detectors (GMX45P4-76, ORTEC, and GCW7023, CANBERRA). We used both Gamma Studio (SEIKO EG&G, Japan) and Spectrum Explorer (CANBERRA, JAPAN) software to analyze the gamma ray spectra. A standard source (MX033U8PP, The Japan Radioisotope Association) was used for the efficiency calibration. The measured radioactivity levels of ^134^Cs and ^137^Cs were corrected for radioactive decay to obtain values for the sampling date. Values under the detection limits were treated as 0 Bq/kg.

### Statistics

Values are presented as means, if normally distributed, and presented as median, if non-normally distributed. The Kruskal-Wallis test or Mann-Whitney’s U-test (2 tailed) were used for statistical analyses with SAS 9.4 (SAS Institute Inc., Cary, NC, USA). Differences with *P* < 0.05 were regarded as statistically significant. Sample sizes are listed in data tables, and in Supplemental Table S1. Sample information of examined mice for radiocesium concentration measurements are presented in Supplemental Table S2.

## Additional Information

**How to cite this article**: Okano, T. *et al.* Effects of environmental radiation on testes and spermatogenesis in wild large Japanese field mice (*Apodemus speciosus*) from Fukushima. *Sci. Rep.* **6**, 23601; doi: 10.1038/srep23601 (2016).

## Supplementary Material

Supplementary Information

## Figures and Tables

**Table 1:**
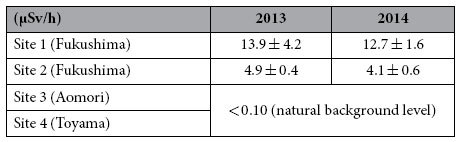
The average ambient dose rates of gamma radiation at ground level for each capture site.

For each site and year, dose rates were measured at 5 spots (different spots in November 2013 and August 2014).

Values are presented as mean ± standard deviation.

**Table 2:** The radioactivity concentrations of ^134^Cs and ^137^Cs in soil.

Bq/kg wet weight)	2013		2014	
	^134^Cs	^137^Cs	^134^Cs	^137^Cs
Site 1 (Fukushima)	72,744 ± 31,677	159,528 ± 79,098	83,924 ± 74,013	218,212 ± 182,773
Site 2 (Fukushima)	36,486 ± 13,608	78,307 ± 25,130	42,918 ± 21,492	119,310 ± 62,235
Site 3 (Aomori)	No data	No data	0 ± 0	12 ± 7
Site 4 (Toyama)	No data	No data	0 ± 0	25 ± 22

Soil samples (n = 5) were collected in November 2013 and July-August 2014.

Values are presented as mean ± standard deviation.

Values under the detection limits were treated as 0 Bq/kg.

**Table 3:** The radioactivity concentrations of ^134^Cs and ^137^Cs in mice (*Apodemus speciosus*).

(Bq/kg wet weight)	2013			2014		
	^134^Cs	^137^Cs	n	^134^Cs	^137^Cs	n
Site 1 (Fukushima)	2,326 (1,771–4,137)	4,331 (3,335–7,806)	12	4,770 (1,140–12,907)	12,312 (2,874–40,131)	10
Site 2 (Fukushima)	1,380 (882–1,766)	2,815 (1,632–3,761)	4	2,359 (954–8,480)	6,426 (2,456–21,738)	8
Site 3 (Aomori)	0 (0–11)	4 (0–21)	10	0 (0–0)	5 (3–8)	5
Site 4 (Toyama)	0 (0–0)	0 (0–3)	10	0 (0–0)	0 (0–6)	8

Male adult mice in the breeding season (April and July-September) were used for the measurements.

Values are presented as median (minimum-maximum).

Values under the detection limits were treated as 0 Bq/kg.

**Table 4:** Testes characteristics of mice (*Apodemus speciosus*) during the breeding season.

	2013				2014			
	Fukushima		Aomori	Toyama	Fukushima		Aomori	Toyama
	Site 1 (12)	Site 2 (4)	Site 3 (25)	Site 4 (17)	Site 1 (6)	Site 2 (6)	Site 3 (5)	Site 4 (6)
Male germ cell apoptosis frequency*	6.8 (0.9–22.8)	7.7 (3.9–12.4)	8.0 (2.5–20.0)	8.3 (1.1–19.5)	5.5 (2.4–9.3)	6.1 (4.2–14.9)	9.2 (6.1–12.0)	9.1 (3.2–16.4)
Seminiferous tubule diameter (μm)	170 ± 15	166 ± 14	159 ± 12	168 ± 11	170 ± 11	169 ± 12	162 ± 20	165 ± 7

^*^Male germ cell apoptosis frequencies are presented as (number of TUNEL positive cells)/(cross sectional area, mm^2^).

Values of male germ cell apoptosis frequency are presented as median (minimum - maximum). Values of seminiferous tubule diameter are presented as mean ± standard deviation. The numbers in parentheses beside site numbers indicate the number of samples used. There were no significant differences in apoptosis rates and seminiferous tubule diameters among different sites and years (Kruskal-Wallis test, *P* > 0.05).

**Table 5:** The frequencies of morphologically abnormal epididymal spermatozoa in wild mice (*Apodemus speciosus*) during the breeding season.

	2013				2014			
	Fukushima		Aomori	Toyama	Fukushima		Aomori	Toyama
	Site 1 (14)	Site 2 (5)	Site 3 (27)	Site 4 (20)	Site 1 (10)	Site 2 (8)	Site 3 (5)	Site 4 (8)
Abnormal head frequency (%)	0 (0–1)	1 (0–2)	0 (0–3)	1 (0–3)	0 (0–1)	0 (0–4)	0 (0–1)	0 (0–2)
Abnormal midpiece frequency (%)	14 (6–55)	12 (8–29)	13 (6–25)	15 (3–33)	15 (12–34)	18 (4–37)	19 (12–30)	17 (5–22)
Abnormal tail frequency (%)	3 (0–14)	4 (0–8)	4 (0–10)	5 (1–14)	4 (1–13)	5 (1–19)	1 (1–8)	5 (1–17)
Normal morphology frequency (%)	84 (40–92)	85 (66–87)	85(72–91)	82 (61–91)	82 (53–87)	78 (57–91)	78 (69–87)	79 (66–94)

Values are presented as median (minimum - maximum).

Numbers in parentheses beside site numbers indicate the number of samples used.

There were no significant differences among sites and years in the frequencies of sperm with head, midpiece, or tail abnormalities, or the frequency of morphologically normal sperm (Kruskal-Wallis test, *P* > 0.05).

## References

[b1] Tokyo Electric Power Company. Estimation of the amount of radioactive materials discharge to the air from the Fukushima Daiichi Nuclear Power Plant (May 2012), http://www.tepco.co.jp/cc/press/betu12_j/images/120524j0105.pdf (Accessed: 21 May 2015) (in Japanese).

[b2] United Nations Scientific Committee on the Effects of Atomic Radiation. Sources and Effects of Ionizing radiation, UNSCEAR 2008 Report to the General Assembly, with scientific annexes, Volume II: Scientific Annex E: Effects of ionizing radiation on non-human biota, http://www.unscear.org/docs/reports/2008/11-80076_Report_2008_Annex_E.pdf (Accessed: 21 May 2015).

[b3] International Commission on Radiological Protection. Environmental Protection: The Concept and Use of Reference Animals and Plants. ICRP Publication 108 (2009).

[b4] Hiyama A. *et al.* The biological impacts of the Fukushima nuclear accident on the pale grass blue butterfly. Sci. Rep. 2, 570 (2012). 22880161 10.1038/srep00570 PMC3414864

[b5] Møller A. P. *et al.* Abundance of birds in Fukushima as judged from Chernobyl. Environ. Pollut. 164, 36–39 (2012). 22321986 10.1016/j.envpol.2012.01.008

[b6] Ochiai K. *et al.* Low blood cell counts in wild Japanese monkeys after the Fukushima Daiichi nuclear disaster. Sci. Rep. 4, 5793 (2014). 25060710 10.1038/srep05793 PMC5376057

[b7] Murase K., Murase J., Horie R. & Endo K. Effects of the Fukushima Daiichi nuclear accident on goshawk reproduction. Sci. Rep. 5, 9405 (2015). 25802117 10.1038/srep09405 PMC4371089

[b8] Geras’kin S. A., Fesenko S. V. & Alexakhin R. M. Effects of non-human species irradiation after the Chernobyl NPP accident. Environ. Int. 34, 880–897 (2008). 18234336 10.1016/j.envint.2007.12.012

[b9] Nakata K., Saitoh T. & Iwasa M. A. Apodemus speciosus (Temminck, 1844) in The Wild Mammals of Japan (eds. Ohdachi S. D., Ishibashi Y., Iwasa M. A. & Saitoh T.) 169–171 (Shoukadoh, 2009).

[b10] Murakami O. Growth and development of the Japanese wood mouse (*Apodemus speciosus*) I. The breeding season in the field. Jpn. J. Ecol. 24, 194–206 (1974) (in Japanese with English summary).

[b11] Endo H. & Yoshiyuki M. Seminiferous tubules of the Japanese wood mouse (*Apodemus speciosus*) in Abukuma Mountains (Fukushima Prefecture). *Mem. Natn. Sci. Mus*., Tokyo 29, 147–151 (1996).

[b12] Okano T. *et al.* Classification of the spermatogenic cycle, seasonal changes of seminiferous tubule morphology and estimation of the breeding season of the large Japanese field mouse (*Apodemus speciosus*) in Toyama and Aomori prefectures, Japan. J. Vet. Med. Sci. 77, 799–807 (2015). 25754934 10.1292/jvms.14-0411 PMC4527501

[b13] Hikida T. & Murakami O. Age determination of the Japanese wood mouse, Apodemus speciosus. Jpn. J. Ecol. 30, 109–116 (1980) (in Japanese with English summary).

[b14] Tatsukawa K. & Murakami O. On the food utilization of the Japanese wood mouse *Apodemus speciosus* (Mammalia: Muridae). Physiol. ecol. Japan 17, 133–144 (1976) (in Japanese with English summary).

[b15] Kondo T. Food habits of the red fox (*Vulpes vulpes japonica*) and the Japanese marten (*Martes melampus melampus*). Bull. Osaka Kyoiku Univ. 29, 19–23 (1980).

[b16] Morii R. & Shioiri T. On the pellet contents of the Ural Owl, *Strix uralensis hondoensis*. Kagawa Seibutsu 23, 15–20 (1996) (in Japanese with English summary).

[b17] Withers H. R., Hunter N., Barkley H. T. & Reid B. O. Jr. Radiation survival and regeneration characteristics of spermatogenic stem cells of mouse testis. Radiat. Res. 57, 88–103 (1974). 10874929

[b18] Meistrich M. L. *et al.* Gradual regeneration of mouse testicular stem cells after exposure to ionizing radiation. Radiat. Res. 74, 349–362 (1978). 149333

[b19] Garnier-Laplace J., Beaugelin-Seiller K. & Hinton T. G. Fukushima wildlife dose reconstruction signals ecological consequences. Environmental science & technology 45, 5077–5078 (2011). 21604757 10.1021/es201637c

[b20] Hashimoto S. *et al.* Predicted spatio-temporal dynamics of radiocesium deposited onto forests following the Fukushima nuclear accident. Sci. Rep. 3, 2564 (2013). 23995073 10.1038/srep02564 PMC3759142

[b21] Kubota Y. *et al.* Estimation of absorbed radiation dose rates in wild rodents inhabiting a site severely contaminated by the Fukushima Dai-ichi nuclear power plant accident. J. Environ. Radioact. 142, 124–131 (2015). 25666988 10.1016/j.jenvrad.2015.01.014

[b22] Hasegawa M. *et al.* Radiocesium concentrations in epigeic earthworms at various distances from the Fukushima Nuclear Power Plant 6 months after the 2011 accident. J. Environ. Radioact. 126, 8–13 (2013). 23933081 10.1016/j.jenvrad.2013.06.006

[b23] Yamanishi *et al.* Survey of environmental radiation in Kawamata-machi, Fukushima-Prefecture. Radioisotopes 62, 259–268 (2013) (in Japanese).

[b24] Watanabe *et al.* Radioactive Cs accumulations in wildlife (some species of birds and mammals) collected from eastern part of Nihonmatsu City, Fukushima Prefecture, Japan. Journal of the Society for Remediation of Radioactive Contamination in the Environment 2, 241–250 (2014) (in Japanese with English summary).

[b25] Tagami K. & Uchida S. *Radiocesium Concentration Change in Game Animals: Use of Food Monitoring Data-13168*. http://www.wmsym.org/archives/2013/papers/13168.pdf (Accessed: 21 May 2015).

[b26] Nakatani J. *et al.* Radionuclide contamination in mammals—future research and countermeasures—. *Honyurui Kagaku* (*Mammalian* Science) 53, 193–196 (2013) (in Japanese).

[b27] Shioya K. Large Japanese field mouse and small Japanese field mouse in *The Encyclopaedia of Animals in Japan, Volume 1: Mammals I* (ed. Kawamichi T.) 94–97 (Heibonsha, 1996) (in Japanese).

[b28] Batzli G. O. Dynamics of small mammal populations: a review in Wildlife 2001: Populations (eds McCullough D. R. *et al.*) 831–850 (Springer, 1992).

[b29] Sanderson G. C. The study of mammal movements: a review. J. Wildl. Manage. 30, 215–235 (1966).

[b30] Dewey W. C., Ling C. C. & Meyn R. E. Radiation-induced apoptosis: relevance to radiotherapy. Int. J. Radat. Oncol. Biol. Phys. 33, 781–796 (1995). 10.1016/0360-3016(95)00214-8 7591884

[b31] Delbès G., Hales B. F. & Robaire B. Toxicants and human sperm chromatin integrity. Mol. Hum. Reprod. 16, 14–22 (2010). 19812089 10.1093/molehr/gap087

[b32] Aitken R. J. & De Iuliis G. N. On the possible origins of DNA damage in human spermatozoa. Mol. Hum. Reprod. 16, 3–13 (2010). 19648152 10.1093/molehr/gap059

[b33] Liu G. *et al.* Effect of low-level radiation on the death of male germ cells. Radiat. Res. 165, 379–389 (2006). 16579650 10.1667/rr3528.1

[b34] Print C. G. & Loveland K. L. Germ cell suicide: new insights into apoptosis during spermatogenesis. BioEssays 22, 423–430 (2000). 10797482 10.1002/(SICI)1521-1878(200005)22:5<423::AID-BIES4>3.0.CO;2-0

[b35] Yamashiro H. *et al.* Effects of radioactive caesium on bull testes after the Fukushima nuclear plant accident. Sci. Rep. 3, 2850 (2013). 24100305 10.1038/srep02850 PMC3792411

[b36] Pomerantseva M. D., Ramaiya L. K. & Chekhovich A. V. Genetic disorders in house mouse germ cells after the Chernobyl catastrophe. Mutat. Res. 381, 97–103 (1997). 9403035 10.1016/s0027-5107(97)00155-3

[b37] Hermosell I. G. *et al.* Patterns of sperm damage in Chernobyl passerine birds suggest a trade-off between sperm length and integrity. Biol. Let. 9, 20130530 (2013). 24088561 10.1098/rsbl.2013.0530 PMC3971692

[b38] Gong E. J. *et al.* Low-dose-rate radiation exposure leads to testicular damage with decreases in DNMT1 and HDAC1 in the murine testis. J. Radiat. Res. 55, 54–60 (2014). 24027299 10.1093/jrr/rrt090 PMC3885123

[b39] Chesser R. K. *et al.* Concentrations and dose rate estimates of ^134, 137^cesium and ^90^strontium in small mammals at Chornobyl, Ukraine. Environ. Toxicol. Chem. 19, 305–312 (2000).

[b40] Beresford N. A. *et al.* Estimating the exposure of small mammals at three sites within the Chernobyl exclusion zone–a test application of the ERICA Tool. J. Environ. Radioact. 99, 1496–1502 (2008). 18450342 10.1016/j.jenvrad.2008.03.002

[b41] Bruce W. R., Furrer R. & Wyrobek A. J. Abnormalities in the shape of murine sperm after acute testicular X-irradiation. Mutat. Res. 23, 381–386 (1974). 4407800 10.1016/0027-5107(74)90112-2

[b42] Rao D. V. *et al.* Induction of sperm head abnormalities by incorporated radionuclides: dependence on subcellular distribution, type of radiation, dose rate, and presence of radioprotectors. Radiat. Res. 125, 89–97 (1991). 1986404 PMC5397899

[b43] Borg C. L. *et al.* Phenotyping male infertility in the mouse: how to get the most out of a ‘non-performer’. Hum. Reprod. Update 16, 205–224 (2010). 19758979 10.1093/humupd/dmp032 PMC2816191

[b44] Cassuto N. G. *et al.* Correlation between DNA defect and sperm-head morphology. Reprod. Biomed. Online 24, 211–218 (2012). 22227364 10.1016/j.rbmo.2011.10.006

[b45] Møller A. P., Surai P. & Mousseau T. A. Antioxidants, radiation and mutation as revealed by sperm abnormality in barn swallows from Chernobyl. Proc. Biol. Sci. 272, 247–253 (2005). 15705548 10.1098/rspb.2004.2914 PMC1634966

[b46] Møller A. P. *et al.* Impaired swimming behaviour and morphology of sperm from barn swallows *Hirundo rustica* in Chernobyl. Mutat. Res. 650, 210–216 (2008). 18218334 10.1016/j.mrgentox.2007.12.006

[b47] Shin S. C., Kang Y. M., Jin Y. W. & Kim H. S. Relative morphological abnormalities of sperm in the caudal epididymis of high-and low-dose-rate γ-irradiated ICR mice. J. Radiat. Res. 50, 261–266 (2009). 19531924 10.1269/jrr.09005

[b48] Woon J. H., Shin S. C., Kang Y. M. & Kim H. S. Sperm abnormalities in high-and low-dose-rate Γ-irradiated Korean dark-striped field mice, *Apodemus agrarius coreae*. Radiat. Prot. Dosimetry 146, 280–282 (2011). 21729943 10.1093/rpd/ncr170

[b49] Committee of Reviewing Taxon Names and Specimen Collections, the Mammal Society of Japan. Guideline for using wild mammals. *Mamm. Sci.* 49, 303-319 (2009) (in Japanese).

