# Supplementary material for: Effects of environmental radiation on testes and spermatogenesis in wild large Japanese field mice (Apodemus speciosus) from Fukushima
Source: Sci Rep. 2016 Mar 23;6:23601. doi: 10.1038/srep23601 (PMC4804236; doi:10.1038/srep23601)
Supplement: Supplementary Information [file srep23601-s1.pdf]

Scientific Reports

Supplemental information

Title: Effects of environmental radiation on testes and spermatogenesis in wild large Japanese field mice (*Apodemus speciosus*) from Fukushima.

Tsukasa OKANO, Hiroko ISHINIWA, Manabu ONUMA, Junji SHINDO, Yasushi YOKOHATA, Masanori TAMAOKI

Table S1. The number of mice used in each experiment.

	Radioactivity concentration		Germ cell apoptosis, seminiferous diameter		Abnormal morphology of sperm	
	2013	2014	2013	2014	2013	2014
Site 1 (Fukushima)	12	10	12	6	14	10
Site 2 (Fukushima)	4	8	4	6	5	8
Site 3 (Aomori)	10	5	25	5	27	5
Site 4 (Toyama)	10	8	17	6	20	8

Table S2. Information on radiocesium (^{134}Cs and ^{137}CS) concentration measurements in the examined mice

Capture Site	Capture month, year	Sample number	Radiocesium concentration (Bq/kg) Median (minimum – maximum)
Site 1 (Fukushima)	Jul, 2013	2	6,954 – 7,637
	Aug, 2013	9	6,392 (5,106 – 11,943)
	Sep, 2013	1	6,624
	Aug, 2014	8	17,082 (4,014 – 54,038)
Site 2 (Fukushima)	Jul, 2013	1	2,514
	Aug, 2013	2	3,585 – 4,806
	Sep, 2013	1	5,527
	Aug, 2014	8	8,785 (3,409 – 30,218)
Site 3 (Aomori)	Aug, 2013	5	4 (0 – 33)
	Sep, 2013	5	4 (3 – 6)
	Apr, 2014	3	8 (3 – 8)
	Aug, 2014	2	4 – 5
Site 4 (Toyama)	Aug, 2013	10	0 (0 – 3)
	Jul, 2014	1	2
	Aug, 2014	7	0 (0 – 6)
